# Supplementary material for: Validating quantitative PCR assays for cfDNA detection without DNA extraction in exercising SLE patients
Source: Sci Rep. 2021 Jun 30;11:13581. doi: 10.1038/s41598-021-92826-4 (PMC8245561; doi:10.1038/s41598-021-92826-4)
Supplement: Supplementary file 1 — Supplementary Information. [file 41598_2021_92826_MOESM1_ESM.pdf]

Validating quantitative PCR assays for cfDNA detection without DNA extraction in exercising SLE patients

Elmo W.I. Neuberger[1], Alexandra Brahmer[1], Tobias Ehlert[1], Katrin Kluge[1], Keito F.A. Philippi[1], Simone C. Boedecker[2], Julia Weinmann-Menke[2] and Perikles Simon[1]

[1]Department of Sports Medicine, Rehabilitation and Disease Prevention, University of Mainz, Mainz, Germany. Albert-Schweitzer Str. 22, 55128 Mainz, Germany.
[2]Department of Rheumatology and Nephrology, University Medical Center Mainz, Germany. Langenbeckstr. 1, 55131 Mainz, Germany.

Table of content:

- Supplementary Table S1
- Supplementary Table S2
- Supplementary Table S3
- Supplementary Figure S1
- Supplementary Table S4
- Supplementary Figure S2
- Supplementary Figure S3
- Supplementary Table S5
- Supplementary Table S6

Supplementary Table S1: Utilized blood collection devices, plastic ware, pipettes and chemicals for the qPCR assays.

Blood collection devices, Plastic ware, and pipetts	Vendor	Cat #
Safety-Lancet: Safety-Lanzette Extra 21G	SARSTEDT	85.1016
Microvette® CB 300 K2E	SARSTEDT	16.444
S-Monovette® 7.5 ml, K3 EDTA, 92x15 mm	SARSTEDT	1605001
Safety-Multifly®-Needle 21G tube 80mm	SARSTEDT	85.1638.203
Filter tips "Avantgµard" 10 µl XL, sterile, surface optimized, with extremely fine tip	Axon Labortechnik	23213
FrameStar® 384-well PCR plates (white wells)	Bio-Budget	34-480LC-0384
AMPLIseal™ Transparent adhesive sealer for RT PCR	Greiner Bio-One	676040
Reaction tubes 0.2	Greiner Bio-One	683201
Reaction tubes 0.5	Greiner Bio-One	667201
Reaction tubes 1.5 ml	Greiner Bio-One	616201
ErgoOne® Single-Channel Pipette, 0.1 – 2.5 µl	STARLAB	S7100-0125
Eppendorf Reference® 2 Ein-Kanal, fix, 20 µL	eppendorf	EP4921000060

Chemicals for qPCR	Vendor	Cat #
UltraPure™ DNase/RNase-Free Distilled Water	Invitrogen™	10977049
VELOCITY DNA Polymerase (500 Units)	BioCat	BIO-21099-BL
5x Hi-Fi Buffer (provided with polymerase)	BioCat	
dNTP Mix (40mM Final Conc.)	BioCat	BIO-39043-BL
SYBR Green nucleic acid gel stain 10,000 x	Sigma	C90M1158

Supplementary Table S2: Sequence of the custom made L1PA2 DNA fragment (GRCh38/hg38_ chr4:68,085,016-68,085,410 / size = 395 bp / strand = +)

Sequence (5´ → 3´)
gaattcAGAATGATGATTTCCAATTTCATCCATGTCCCTACAAAGGACATGAACTCATCAT<br>TTTTTATGGCTGCATAGTATTCCATGGTGTATATGTGCCACATTTTCTTAATCCAGTCT<br>ATCATTGTTGGACATTTGGATTGGTTCCAAGTCTTTGCTATTGTGAATAA***TGCCGCAAT***<br>***AAACATACGTG***TGCATGTGTCTTTATAGCAGCATGATTTATAGTCATTTGGGTATATAC<br>CCA***GTAATGGGATGGCTGGGTC***AAATGGTATCTCTAGTTCTAGATCCCTGAGGAATC<br>GCCACACTGACTTCCACAATGGTTGAACTAGTTTACAGTCCCACCAACAGTGTAAAAG<br>TGTTCCTATTTCTCCACAT***CCTCTCCAGCACCTGTTGTT***TCCTGACTTgaattc

The binding site for the L1PA2_fw primer is highlighted in grey. The binding sites for the reverse primers of the L1PA2_90bp and L1PA2_222bp assays are highlighted in blue and green, respectively.

Supplementary Table S3: Primer sequences

L1PA2 PCR Primers [0.14 µmol/PCR]	Sequence in 5’ → 3’	Amplicon size	Annealing temperature
L1PA2_fw	TGCCGCAATAAACATACGTG		60.4 °C
L1PA2_90bp_rv	GACCCAGCCATCCCATTAC	90 bp	61.1 °C
L1PA2_222bp_rv	AACAACAGGTGCTGGAGAGG	222 bp	63.4 °C

Supplementary Figure S1: Distribution of the hits per chromosome in the human genome (GRCh38/hg38). The figure was produced using Microsoft Office Excel 2016 (Microsoft Corp., Redmond, WA, USA).

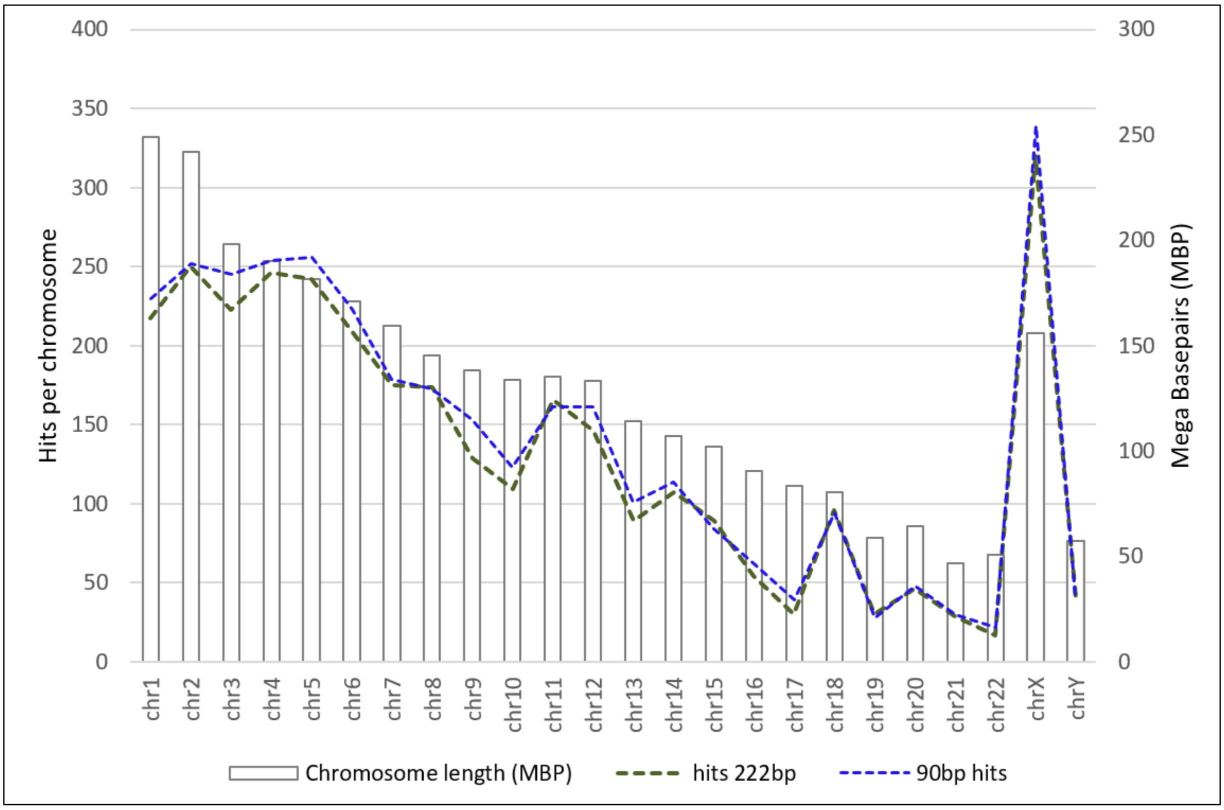

Supplementary Table S4:

Predicted targets from the *UCSC In-Silico PCR* (length and number) for the L1PA2_fw and L1PA2_222bp_rv primer pair in GRCh38/hg38. The Max Product Size (maximum size of amplified region) was set to 660 bp, which equals the predicted maximal amplification rate of the polymerase during 10s amplification. Min Perfect Match (number of bases that match exactly on 3' end of primers) was set to 19 bp.

**L1PA2_90bp**	
Sequence length	Hits in the Genome
90bp	3338
89bp	28
91bp	14
92bp	7
88bp	5
87bp	4
68bp	2
79bp	2
321bp	2
96bp	2
330bp	1
422bp	1
94bp	1
278bp	1
186bp	1
76bp	1
97bp	1
260bp	1
86bp	1
81bp	1
95bp	1
**Total**	**3416**

**L1PA2_222bp**	
Sequence length	Hits in the Genome
222bp	2985
221bp	117
223bp	35
220bp	20
219bp	10
224bp	10
226bp	5
228bp	4
212bp	4
227bp	4
218bp	3
213bp	2
210bp	2
200bp	2
217bp	2
209bp	2
204bp	2
240bp	2
230bp	2
216bp	2
232bp	2
197bp	1
235bp	1
236bp	1
225bp	1
410bp	1
229bp	1
215bp	1
408bp	1
205bp	1
392bp	1
233bp	1
214bp	1
242bp	1
238bp	1
190bp	1
211bp	1
558bp	1
208bp	1
203bp	1
256bp	1
**Total**	**3237**

Supplementary Figure S2: Typical amplification curves of the reference samples and H_2O or mouse plasma NTCs of the L1PA2_90bp assay (A), and corresponding melt curves (B). Typical L1PA2_222bp amplification curves of the reference samples with NTC (C), and corresponding melt curves (D). RFU = relative fluorescence units. The reference samples are PRE- POST-exercise plasma samples from a single subject, diluted 1:10 in H_2O. The figure was produced using the Bio-Rad CFX Manager software Version: 3.1.1517.0823.

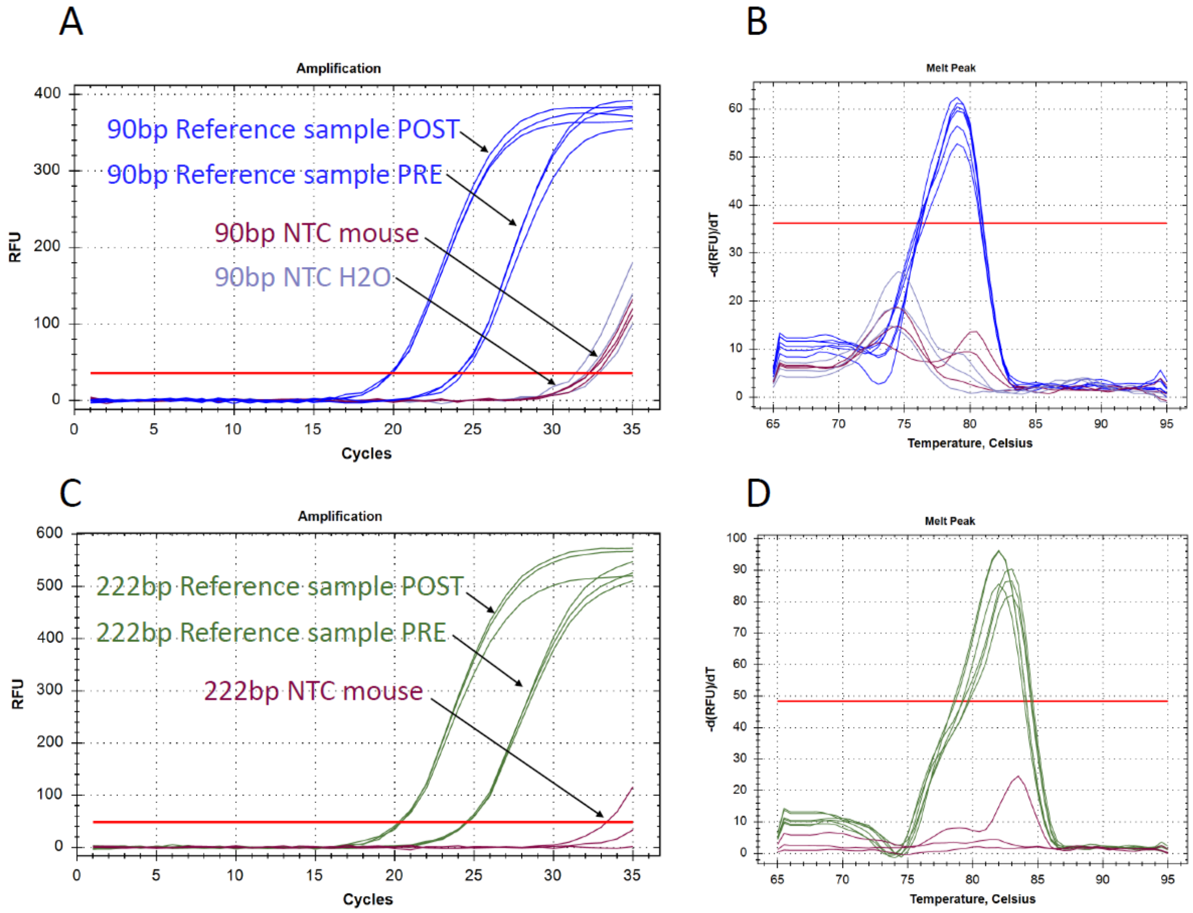

Supplementary Figure S3: cfDNA concentrations after extended storage of blood samples. The figure was produced using Microsoft Office Excel 2016 (Microsoft Corp., Redmond, WA, USA).

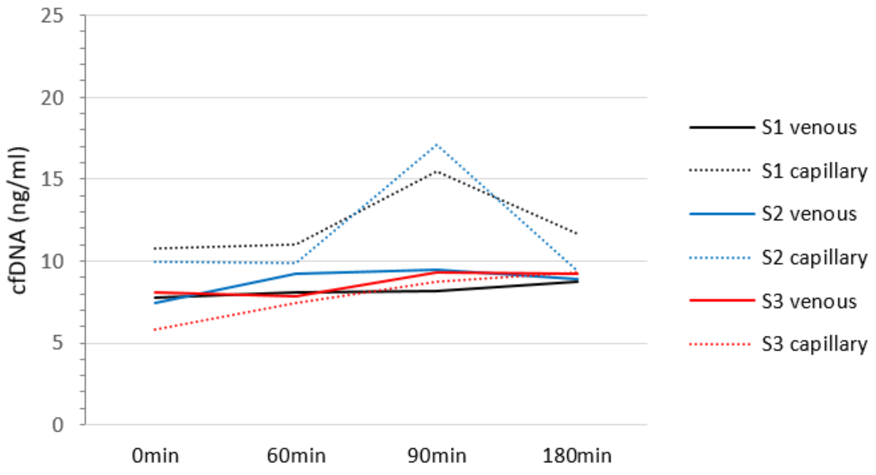

After prolonged storage of the blood samples before centrifugation (60min, 90 min, or 180 min) the cfDNA concentrations do not differ relevantly in three subjects. Capillary cfDNA samples show a higher variance compared to venous samples.

Supplementary Table S5: Results of the post-hoc comparisons between capillary and venous samples, related to Linear mixed model fit by REML ['lmerMod'].

Formula: log10(cfDNA_ng_ml) ~ Timepoint * Sample_type + (1 | Subject)

```
Timepoint Sample_type response   SE   df lower.CL upper.CL
 +90 min   capillary       13.6 1.49 44.9     10.0     18.4
 POST      capillary       24.8 2.69 43.0     18.3     33.4
 PRE       capillary       11.1 1.20 43.0      8.2     14.9
 +90 min   venous          14.1 1.53 42.1     10.5     19.0
 POST      venous          29.6 3.20 42.1     22.0     39.9
 PRE       venous          13.9 1.51 42.1     10.4     18.8

Degrees-of-freedom method: kenward-roger
Confidence level used: 0.95
Conf-level adjustment: sidak method for 6 estimates
Intervals are back-transformed from the log10 scale

$contrasts
 contrast                                estimate     SE  df t.ratio p.value
 (+90 min capillary) - POST capillary   -0.26040 0.0345 130  -7.544 <.0001
 (+90 min capillary) - PRE capillary     0.08916 0.0345 130   2.583 0.1089
 (+90 min capillary) - (+90 min venous) -0.01662 0.0342 130  -0.487 0.9966
 (+90 min capillary) - POST venous      -0.33834 0.0342 130  -9.906 <.0001
 (+90 min capillary) - PRE venous       -0.01128 0.0342 130  -0.330 0.9995
 POST capillary - PRE capillary          0.34956 0.0337 130  10.367 <.0001
 POST capillary - (+90 min venous)       0.24378 0.0334 130   7.308 <.0001
 POST capillary - POST venous           -0.07794 0.0334 130  -2.337 0.1871
 POST capillary - PRE venous             0.24912 0.0334 130   7.468 <.0001
 PRE capillary - (+90 min venous)       -0.10578 0.0334 130  -3.171 0.0228
 PRE capillary - POST venous            -0.42750 0.0334 130 -12.816 <.0001
 PRE capillary - PRE venous             -0.10044 0.0334 130  -3.011 0.0362
 (+90 min venous) - POST venous         -0.32172 0.0330 130  -9.750 <.0001
 (+90 min venous) - PRE venous           0.00534 0.0330 130   0.162 1.0000
 POST venous - PRE venous                0.32706 0.0330 130   9.912 <.0001

Note: contrasts are still on the log10 scale
Degrees-of-freedom method: kenward-roger
P value adjustment: tukey method for comparing a family of 6 estimates
```

Supplementary Table S6: Results of the post-hoc comparisons between capillary samples at all timepoints, related to Linear mixed model fit by REML ['lmerMod'].

Formula: log10(cfDNA_ng_ml) ~ Timepoint + (1 | Subject)

Timepoint	response	SE	df	lower.CL	upper.CL
+15 min	24.7	2.87	45.8	17.83	34.2
+30 min	22.5	2.63	46.9	16.18	31.2
+60 min	15.1	1.76	46.8	10.86	20.9
+90 min	13.6	1.61	49.1	9.76	18.9
POST	24.8	2.90	46.8	17.87	34.4
PRE	11.1	1.30	46.8	8.00	15.4
RQ	16.5	1.92	45.8	11.92	22.9

```
Degrees-of-freedom method: kenward-roger
Confidence level used: 0.95
Conf-level adjustment: sidak method for 7 estimates
Intervals are back-transformed from the log10 scale
```

$contrasts

contrast	estimate	SE	df	t.ratio	p.value
(+15 min) - (+30 min)	0.04137	0.0382	155	1.082	0.9325
(+15 min) - (+60 min)	0.21461	0.0382	155	5.616	<.0001
(+15 min) - (+90 min)	0.25918	0.0391	155	6.627	<.0001
(+15 min) - POST	-0.00167	0.0382	155	-0.044	1.0000
(+15 min) - PRE	0.34729	0.0382	155	9.088	<.0001
(+15 min) - RQ	0.17489	0.0378	155	4.626	0.0002
(+30 min) - (+60 min)	0.17325	0.0386	155	4.485	0.0003
(+30 min) - (+90 min)	0.21781	0.0394	155	5.530	<.0001
(+30 min) - POST	-0.04303	0.0386	155	-1.114	0.9230
(+30 min) - PRE	0.30593	0.0386	155	7.920	<.0001
(+30 min) - RQ	0.13352	0.0382	155	3.493	0.0109
(+60 min) - (+90 min)	0.04456	0.0395	155	1.128	0.9187
(+60 min) - POST	-0.21628	0.0386	155	-5.601	<.0001
(+60 min) - PRE	0.13268	0.0386	155	3.436	0.0131
(+60 min) - RQ	-0.03972	0.0382	155	-1.040	0.9440
(+90 min) - POST	-0.26084	0.0395	155	-6.601	<.0001
(+90 min) - PRE	0.08812	0.0395	155	2.230	0.2858
(+90 min) - RQ	-0.08429	0.0391	155	-2.155	0.3263
POST - PRE	0.34896	0.0386	155	9.036	<.0001
POST - RQ	0.17655	0.0382	155	4.620	0.0002
PRE - RQ	-0.17240	0.0382	155	-4.512	0.0003

```
Note: contrasts are still on the log10 scale
Degrees-of-freedom method: kenward-roger
P value adjustment: tukey method for comparing a family of 7 estimates
```
